# Baseline mitochondrial DNA copy number and heart failure incidence and its role in overall and heart failure mortality in middle-aged women

**DOI:** 10.3389/fcvm.2022.1012403

**Published:** 2022-11-10

**Authors:** Kristina Sundquist, Jan Sundquist, Xiao Wang, Karolina Palmer, Ashfaque A. Memon

**Affiliations:** Center for Primary Health Care Research, Lund University/Region Skåne, Malmö, Sweden

**Keywords:** mitochondrial copy number, mitochondrial dysfunction, risk assessment, heart failure, mortality

## Abstract

Heart failure (HF) is a leading cause of death in both men and women. However, risk factors seem to differ for men and women and significant gaps in sex-specific knowledge exist. Mitochondria are critical for cardiomyocytes and in this study, we investigated the role of baseline mitochondrial DNA copy number (mtDNA-CN) in HF incidence in middle-aged women and its possible role in the association between myocardial infarction (MI) and HF. Finally, we also investigated whether baseline mtDNA-CN was associated with overall and HF mortality. Baseline levels of mtDNA-CN were quantified by droplet digital PCR in a population-based follow-up study of middle-aged (50–59 years) Swedish women (*n* = 2,508). The median follow-up period was 17 years. Levels of mtDNA-CN were associated with age, BMI, alcohol, smoking, education, physical activity and lipid biomarkers. Multivariable Cox regression analysis adjusted for potential confounders showed that each standard deviation decrease of baseline mtDNA-CN was associated with higher incidence of HF (HR = 1.34; 95% CI=1.11–1.63). Similar results were obtained when mtDNA-CN levels were categorized into quartiles with lowest vs. highest quartile showing the highest risk of HF incidence (HR = 2.04 95% CI=1.14; 3.63). We could not detect any role of mtDNA-CN in the association between MI and HF incidence. Lower baseline mtDNA-CN levels were associated with both overall (HR = 1.27; 95% CI=1.10–1.46) and HF mortality (HR = 1.93; 95% CI=1.04–3.60); however, in multivariable analysis adjusted for potential confounders, the higher risks of HF mortality were no longer significant (HR=1.57; 95% CI=0.85–2.90). In conclusion, low baseline mtDNA-CN is an easily quantifiable molecular risk factor for HF incidence and may be a risk factor for overall and HF-related mortality.

## Introduction

Heart failure (HF) is a growing public health problem characterized by frequent hospitalization, poor quality of life and a higher mortality rate. Despite a decline in the overall risk of cardiovascular diseases (CVD), HF rates have remained unchanged (1). Between the ages of 65 and 85 years, it is estimated that the incidence of HF doubles in men with each 10-year increase, whereas the rate of incidence of HF triples in the same time frame among women (2).

MI is known to be the most common cause of HF in both men and women; however, pathophysiological factors associated with the progression of MI to HF seem to be different in men and women (3). Recent epidemiological studies further support the sex differences for HF type. For example, HF with preserved ejection fraction (HFpEF), which is associated with high morbidity and mortality and lacks any proven therapy, is the most common HF phenotype among women, compared to men who are predisposed to HF with reduced ejection fraction (HFrEF) (4). The higher risk of HFrEF in men compared to women has been attributable to their predisposition to macrovascular coronary heart disease, whereas coronary microvascular dysfunction/endothelial inflammation has been postulated to play a major role in HFpEF (5), which is more common in women. However, initiation and progression of inflammatory responses within the heart remain poorly defined.

Mitochondria are critical for normal functioning of cardiomyocytes and its dysfunction can lead to pathophysiological consequences in heart tissue and beyond (6). Damaged mitochondria are normally removed by autophagy/lysosome system and cardiomyocytes (7). Mitochondrial DNA that escapes autophagy leads to inflammatory responses in cardiomyocytes and may induce myocarditis, and dilated cardiomyopathy (8). Mitochondrial copy number (mtDNA-CN) is a surrogate marker of mitochondrial dysfunction (9) and therefore it can be a useful clinical biomarker for risk assessment of several diseases. However, it is important to note that mtDNA-CN depends on cell-type and the disease in question (10). For example, using whole blood, we and others have shown that mtDNA-CN is decreased in people with CVDs (11) whereas both low and high mtDNA-CN have been shown in people with T2D (12, 13) and cancer (14), probably depending on study design, severity of the disease and methods used.

Nevertheless, mtDNA-CN is easily quantifiable in a large-scale manner due to current high-throughput and accurate methods such as droplet digital PCR. It can thus be used for absolute quantification of mtDNA-CN in blood (15) where higher mtDNA-CN is a biomarker of better mitochondrial function and vice versa. Mitochondrial dysfunction is associated with aging and can affect cellular functions and thereby result in a variety of cardiovascular diseases such as MI, cardiomyopathy, heart failure, and arrhythmias (11, 16, 17). Despite the improvements in therapy, the absolute mortality for HF remains approximately 50% within 5 years after diagnosis (18). It is therefore important to identify novel risk factors and biomarkers that could lead to better treatment approaches, prevention, and risk stratification (19). Even though mitochondria play an important role in normal function and mitochondrial dysfunction is associated with CVD and its risk factors, its role in HF, especially in women, is not well established.

The primary prevention of HF in women should involve targeted, sex-specific strategies, however, despite the sex differences, women are underrepresented (20–25% of cohorts) in most of the clinical and epidemiological studies (5). Furthermore, molecular and clinical risk factors for HF in middle-aged women are not well established. We could find only two studies on the role of mtDNA-CN and HF, an observational study (including 66% males) followed for a median follow-up of only 17 months, demonstrated that lower mtDNA-CN was associated with higher risk of HF (20) and another follow-up study on both men and women showed that lower baseline mtDNA-CN was associated with higher HF incidence (21).

We have recently shown that lower baseline mtDNA-CN levels are associated with MI incidence in middle-aged women (11). In this study, we examined the role of mtDNA-CN in HF incidence and its role in the association between MI and HF. In addition, we also investigated the role of mtDNA-CN in overall and HF mortality. Mitochondrial DNA-CN was quantified in whole blood samples obtained from a well-defined population-based study on middle-aged women with a median follow-up of 17 years. We used a well optimized ddPCR based method for accurate and absolute quantification of mtDNA-CN.

## Materials and methods

### Study population

**Figure 1:**
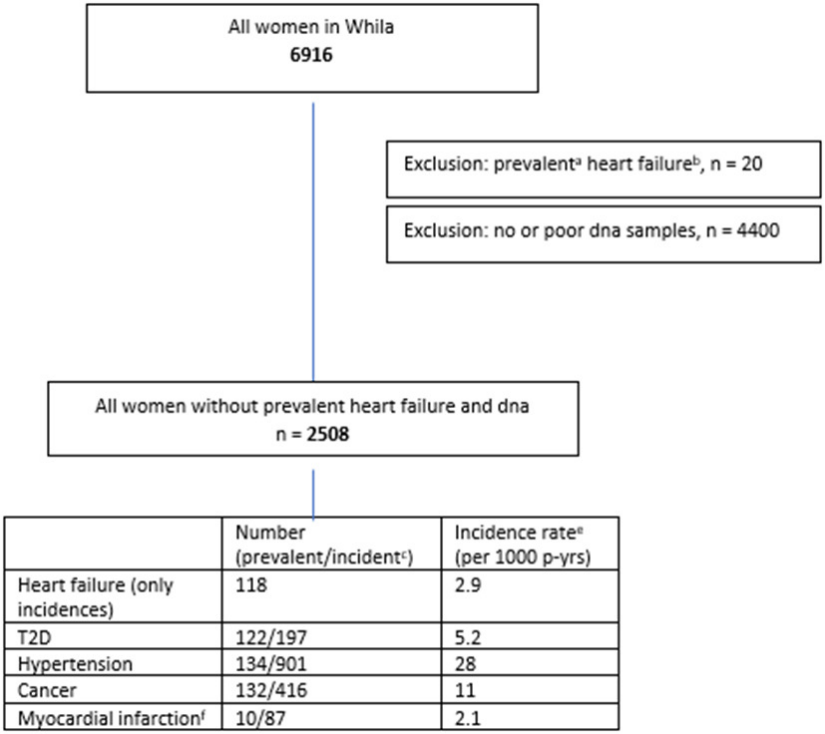
Heart failure diagnoses were found in primary health care register (60%), hospital discharge register (33%), outpatient register (7%) and death register (0.3%). ^a^Prevalent is defined as first diagnose date before or at baseline. ^b^Heart failure icd-codes = I50, I110, 428, 427.00, 427.10, 434.10, and 434.20. ^c^Incident is defined as first diagnose date after baseline. ^d^Cumulative incidence = number of cases/number of individulas at baseline. ^e^Incidence rate = number of cases/total person-years of observation during study. ^f^Mycardial infarction icd-codes = 121, 410, 410, 420.10, and 420.17.

Women health in Lund area (WHILA) is a well-characterized population-based follow-up study. All women aged 50–65 years (born between 1935 and 1945) who living in southern country (Region Skåne) of Sweden were invited to participate in a health survey. From Dec 1995 to Feb 2000, a total of 6,916 women (out of 10,766, the total population of women in in the five southern municipalities in 1995) underwent a physical examination and answered a questionnaire. The questionnaire that was distributed to all participants has been described previously (22). Participants were followed from the day of screening until death, or if no event occurred until May 31st, 2015. The primary end point for the present study is a first occurrence of fatal or non-fatal HF, death from any cause. Participants were followed from the day of screening until primary endpoint or death, or if no event occurred until May 31st, 2015. However, the blood samples for DNA extraction were collected midway through this study (from October 1997), therefore, approximately half of the participants' blood samples were not available for mtDNA-CN quantification and after exclusion of samples with poor quality of DNA and prevalent HF (*n* = 20), 2,508 participants were included in the present study (Figure 1).

### Variables

Age at screening, BMI biomarkers of lipid metabolism, blood pressure and glucose were used as continuous variables. These variables were measured as described previously (23). Educational level was categorized according to the number of years of education as follows: <= 9 years as low, 10–12 years as middle and > 12 years as higher education. Physical activity was defined according to the questionnaire with a score between 1 and 6 into low and high activity. Participants with the score 1–3 were categorized as low activity at home (1= hardly do anything at all, 2= mostly sedentary, 3= light physical exertion). High activity at home was categorized with a score between 4 and 6 (4= strenuous exercise 1–2 h/week, 5= strenuous exercise at least 3 h/week, 6= hard regular exercise).

Alcohol consumption was assessed by the questionnaire as described previously (24). Smoking information was obtained by questionnaire and was categorized as current smokers and no or former smokers.

All explanatory variables, except age, weight and height, MI, HF, T2D and mortality were self-reported in the validated questionnaire (25). Age was taken from the population register, while weight, height, and T2D were obtained from a clinical investigation. MI, HF, and mortality information were collected from the Swedish nationwide and regional registers, which includes, primary health care diagnoses, the hospital discharge register, the outpatient register and the death register. HF cases were found mainly from the primary health care register (60%), followed by the hospital discharge register (33%), outpatient register (7%) and a few cases from the death register (0.3%) (Figure 1).

### Quantification of MtDNA copy number by droplet digital PCR

A full description of the method is provided in the Supplementary information. Briefly, DNA was extracted from whole blood and quantification of mtDNA-CN was performed by droplet digital PCR based method, as also described previously (15) and data were analyzed using QuantaSoft™ Software, which determines the numbers of droplets that were positive and negative for each fluorophore in each sample. The fraction of positive droplets was then fitted to a Poisson distribution in QuantaSoft™ Software to determine the absolute copy number in units of copies/μl. DNA preparation and PCR experiments were performed in separate designated rooms and each run included negative and positive controls. No significant hazards or risks are associated with the reported work.

### Statistical analysis

Prevalent diseases were defined as diagnosed at baseline or before inclusion, while incident disorders were defined as first diagnosis during follow-up. All participants with a mitochondrial DNA measure and no prevalent heart failure (HF) were followed from baseline to incident HF, death or end of study, whichever came first.

We used univariate linear regression models to examine the association between mtDNA-CN and clinical risk factors. To estimate hazard ratios for the association between mtDNA-CN and clinical risk factors and time to incident HF, we used univariate Cox proportional hazards models. The association between mtDNA-CN and HF was then adjusted for the clinical variables significantly associated with both mtDNA-CN and HF, including age, BMI, smoking, education, systolic blood pressure, triglycerides, high-density lipoprotein (HDL) and prevalent type two diabetes (T2D). MtDNA-CN was in these Cox regression models reversed to estimate HR for decrease in mtDNA-CN and standardized to get a comparable scale. We also categorized mtDNA-CN into quartiles where we used the lowest quartile as the reference in the models. The same method was used to examine the association between mtDNA-CN and mortality, both overall and due to HF. In these models the adjusting variables were age, smoking, alcohol consumption, education, physical activity, and HDL. We used Schoenfeld residuals to test the proportionality assumption in the Cox regression models. During the follow-up time, several competing events, which potentially can prevent HF from happening, were defined. To consider the informative censoring nature of these competing events, we therefore also analyzed the association between mtDNA-CN and HF by using competing risk analysis. The competing events were cancer, myocardial infarction (MI) and death. We calculated subdistributional hazard ratios using the method of Fine and Gray (26). We also plotted a cumulative incidence graph that quantifies the probability of HF accounting for competing risks. This graph is shown for quartiles of mtDNA-CN.

To investigate the role of mtDNA-CN and MI in the association with HF we used three different methods. First, we examined the possibility of MI or mtDNA-CN acting as confounders. Second, we used interaction analyses to estimate MI and mtDNA-CN as effect modifiers in the risk of future HF. Last, we examined the mediating effect of MI and mtDNA-CN where we separated the total effect into direct and indirect effects. In order to investigate the robustness of our results we performed a sensitivity analyses after having excluded women with prevalent MI. Statistical analyses were performed by using STATA version 16 (StataCorp LP).

## Results

### Baseline characteristics and MtDNA-CN levels in HF

In total 2,508 women with mtDNA-CN measures and no prevalent HF were included in the study. Participants diagnosed with HF during the follow-up (incidence) were older and had higher BMI, higher prevalence of smoking, lower level of education, higher systolic and diastolic blood pressure, and lower HDL levels. Prevalent T2D, prevalent MI and prevalent cancer were more common in participants with HF than in participants with no HF during the follow-up (Table 1).

**Table 1:** Baseline characteristics for all individuals with a mtDNA-CN measure, stratified by no heart failure and incident heart failure.

	**Total** **(*n* = 2,508)**	**No incident HF** **(*n* = 2,390)**	**Incident HF (*n* = 118)**	***p*** **-value**
MtDNA-CN, median (IQR) Min-max Missing	117 (35) 32.4–226 0%	117 (35) 32.8–226 0%	106 (29) 32.4–178 0%	0.0002
Age, median (IQR) Missing	57 (4) 0%	57 (4) 0%	58 (5) 0%	0.002
BMI, median (IQR) Missing	25 (5) 0%	25 (5) 0%	26 (7) 0%	0.03
Smoker, %				
Yes or former/no Missing	20/80 2%	20/80 2%	27/73 0.9%	0.045
Alcohol consumption^a^, %				
Low/medium/high Missing	26/61/14 5%	25/61/14 5%	33/50/17 7%	0.06
Education, %				
< = 9 years, 10–12, > 12 Missing	55/16/30 1%	54/16/30 2%	69/14/17 0.9%	0.003
Activity at home^b^, %				
Low/medium/high Missing	5/91/4 2%	5/91/5 2%	8/89/3 2%	0.32
Systolic blood pressure, mean (SD) Missing	132 (17) 0.08%	132 (17) 0.08%	138 (20) 0%	0.0001
Diastolic blood pressure, mean (SD) Missing	85 (9.0) 0.04%	85 (8.9) 0.04%	87 (9.1) 0%	0.005
Total cholesterol, mean (SD) Missing	6.0 (1.1) 0.08%	6.0 (1.1) 0.08%	5.9 (1.1) 0%	0.20
Triglycerides, median (IQR) Missing	1.5 (1.0) 0.04%	1.5 (1.0) 0.04%	1.7 (1.1) 0%	0.08
HDL, mean (SD) Missing	1.8 (0.5) 0.04%	1.8 (0.4) 0.04%	1.7 (0.5) 0%	0.03
LDL, mean (SD) Missing	3.4 (1.0) 11%	3.4 (1.0) 11%	3.4 (1.0) 12%	0.91

a Low alcohol = 0 grams per day, medium = 0.1–11.9 and high ≤ 12 grams per day.

b Activity at home was categorized from a 6-point scale.

Low: 1 = hardly anything at all or 2 = mostly sedentary.

Medium: 3 = lighter physical exertion or 4 = strenuous exercise 1–2 h/week.

High: 5 = strenuous exercise at least 3 h/week or 6 = hard regular exercise.

### Association between MtDNA-CN and clinical risk factors

Univariate linear regression analysis was performed to investigate the association between mtDNA-CN and clinical risk factors. Higher levels of mtDNA-CN were inversely associated with age, BMI, smoking, education level, physical activity at home, systolic blood pressure, triglycerides and prevalent T2D. Higher levels of mtDNA-CN were positively associated with alcohol consumption and HDL. Diastolic blood pressure, total cholesterol, LDL, prevalent cancer and prevalent MI were not significantly associated with mtDNA-CN (Table 2).

**Table 2:** Univariate linear regression models examining association between mtDNA-CN and clinical risk factors.

**Outcome: MtDNA–CN**	**β**	***p*** **–value**	**95% CI**
Age at baseline (years)	−0.5	0.01	−0.9; −0.1
BMI (kg/m^2^)	−0.3	0.03	−0.5; −0.03
Smoker (yes or former vs. no)	−8.0	< 0.0001	−0.1; −5.4
Alcohol consumption (grams per day)	2.5	0.006	0.7; 4.2
Education (< = 9 years vs. > 9 years)	−3.4	0.002	−5.5; −1.3
Activity at home (low vs. medium or high)^a^	−8.3	0.001	−13; −3.4
Systolic blood pressure (mmHg)	−0.1	0.02	−0.1; −0.01
Diastolic blood pressure (mmHg)	−0.04	0.52	−0.2; 0.1
Total cholesterol (mg/dL)	0.7	0.18	−0.3; 1.6
Triglycerides (mg/dL)	−1.8	0.001	−2.9; −0.7
HDL (mg/dL)	4.8	< 0.0001	2.5; 7.1
LDL (mg/dL)	0.8	0.14	−0.3; 2.0
Prevalent T2D (yes vs. no)	−7.2	0.004	−12; −2.3
Prevalent cancer (yes vs. no)	2.6	0.28	−2.1; 7.2
Prevalent MI (yes vs. no)	−3.2	0.71	−19; 13

a Low = hardly anything at all or mostly sedentary, medium or high = lighter physical exertion, strenuous exercise 1–2 h/week, strenuous exercise at least 3 h/week or hard regular exercise.

### Association between MtDNA-CN and incident HF and other risk factors

**Figure 2:**
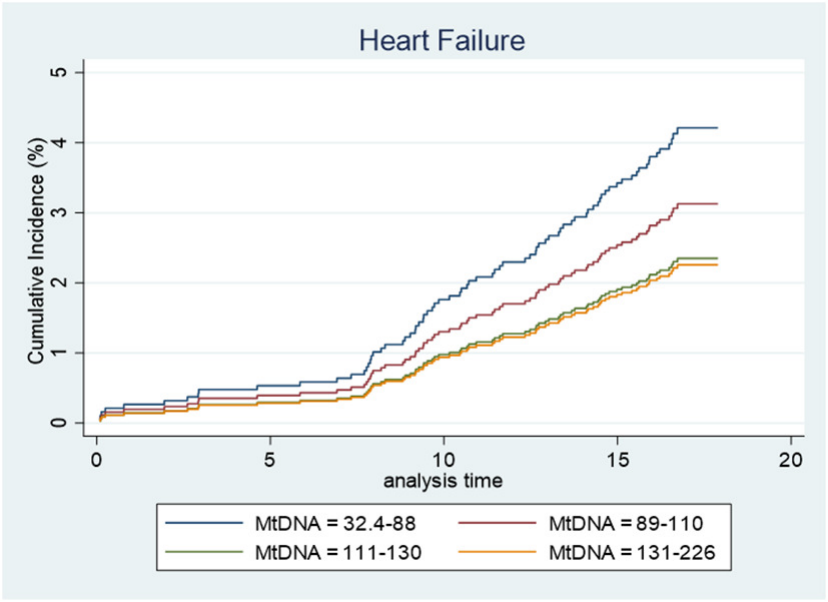
Cumulative incidence function after adjusted (for age, bmi, smoking, education, systolic, triglyceride, and HDL) competing risk regression where cancer, MI, and death are considered competing events.

Unadjusted Cox regression analysis showed that there was a significant association between baseline levels of mtDNA-CN and future risk of HF. The proportionality assumption was tested using Schoenfeld residuals and no violation of the proportionality assumption was found (*p* = 0.62), which justified the use of the hazard models. Low mtDNA-CN (1 standard deviation decrease) was associated with a 44% higher HF incidence (HR = 1.44; 95% CI = 1.20–1.74). Categorization of mtDNA-CN into quartiles showed a dose-dependent effect on the associations between mtDNA-CN and HF. Participants in the lowest quartile of mtDNA-CN had 2.6-times the hazard of developing HF compared to participants in the highest quartile (Table 3). Among risk factors, prevalent MI was the strongest risk factor for HF (HR = 11.2; 95% CI = 4.13–30.3). Age at baseline, BMI, smoking, education level, systolic and diastolic blood pressure, triglycerides and prevalent T2D were also associated with increased risk of HF incidence whereas higher HDL levels were associated with lower HF incidence (Table 3). In the multivariable model, variables which were significantly associated with both mtDNA-CN (Table 2) and HF incidence (Table 3 univariate analysis) were included. Lower mtDNA-CN levels were significantly associated with higher HF incidence even after adjusting for potential confounders. A dose dependent effect was observed when mtDNA-CN levels were stratified in quartiles (Table 3). The potential effect of baseline mtDNA-CN on the probability of HF as first event in the presence of competing events such as cancer, MI and death was analyzed. Cumulative incidence function after adjusting for age, BMI, smoking, education, systolic blood pressure, triglycerides and HDL showed that lower mtDNA-CN levels were associated with higher cumulative HF incidence compared to higher mtDNA-CN (Figure 2). Fine and Gray method was used to calculate subdistribution hazard ratio (SHR) and 1 SD decrease in mtDNA-CN was associated with a 32% increase in SHR (SHR = 1.32; 95% CI = 1.03–1.71) in the presence of competing risks and adjusted for age, BMI, smoking, education, systolic blood pressure, triglycerides and HDL (Table 4).

**Table 3:** Cox regression models examining effect of mtDNA-CN and other risk factors on risk for incident heart failure.

	**Univariate**			**Adjusted** ^a^		
**Outcome: time to heart failure**	**HR**	***p*** **-value**	**95% CI**	**HR**	***p*** **-value**	**95% CI**
MtDNA-CN (decrease, std)^b^	1.44	< 0.0001	1.20; 1.74	1.34	0.003	1.11; 1.63
MtDNA-CN quartiles^c^						
Q_3_ vs. Q_4_	1.44	0.18	0.84; 2.47	1.18	0.56	0.68; 2.04
Q_2_ vs. Q_4_	1.91	0.01	1.14; 3.20	1.63	0.07	0.96; 2.74
Q_1_ vs. Q_4_	2.60	0.001	1.47; 4.58	2.04	0.02	1.14; 3.63
Age at baseline (years)	1.11	0.001	1.04; 1.19			
BMI (kg/m^2^)	1.05	0.02	1.01; 1.09			
Smoker (yes or former vs. no)	1.55	0.04	1.03; 2.32			
Alcohol consumption (grams per day)	0.89	0.46	0.66; 1.21			
Education (< = 9 years vs. > 9 years)	1.90	0.001	1.28; 2.81			
Activity at home (low vs. medium or high)	1.67	0.14	0.85; 3.30			
Systolic blood pressure (mmHg)	1.02	< 0.0001	1.01; 1.03			
Diastolic blood pressure (mmHg)	1.03	0.004	1.01; 105			
Total cholesterol (mg/dL)	0.90	0.22	0.75; 1.07			
Triglycerides (mg/dL)	1.22	0.01	1.05; 1.43			
HDL (mg/dL)	0.63	0.02	0.42; 0.94			
LDL (mg/dL)	1.02	0.87	0.84; 1.24			
Prevalent T2D (yes vs. no)	2.13	0.02	1.14; 3.96			
Prevalent cancer (yes vs. no)	1.96	0.03	1.05; 3.65			
Prevalent MI (yes vs. no)	11.2	< 0.0001	4.13; 30.3			

a Adjusted for age, bmi, smoking, education, systolic, triglycerides, HDL and prevalent T2D.

b MtDNA-CN has been reversed and standardized (HR for a one standard deviation decrease in mtDNA-CN).

c Q_1_ = 32.4–88, Q_2_ = 89–110, Q_3_ = 111–130, Q_4_ = 131–226.

**Table 4:** Effect of mtDNA on heart failure using competing-risks regression model by a subdistribution hazard approach (fine and gray).

	**Univariate**			**Adjusted** ^a^		
**Outcome: time to heart failure**	**SHR^b^**	***p*** **-value**	**95% CI**	**SHR**	***p*** **-value**	**95% CI**
MtDNA (decrease, std)^c^	1.40	0.008	1.09; 1.80	1.32	0.03	1.03; 1.71
MtDNA quartiles^d^						
Q_3_ vs. Q_4_	1.23	0.54	0.64; 2.34	1.04	0.91	0.54; 2.02
Q_2_ vs. Q_4_	1.56	0.16	0.84; 2.91	1.39	0.30	0.74; 2.61
Q_1_ vs. Q_4_	2.34	0.01	1.19; 4.58	1.88	0.07	0.96; 3.71

a Adjusted for age, bmi, smoking, education, systolic, triglycerides and hdl.

b Subdistributional hazard ratio where cancer, MI and death are considered competing events.

c MtDNA has been reversed and standardized (HR for a one standard deviation decrease in mtDNA).

d Q_1_ = 32.4–88, Q_2_ = 89–110, Q_3_ = 111–130, Q_4_ = 131–226.

### The role of MtDNA-CN and MI in the association with incident HF

**Figure 3:**
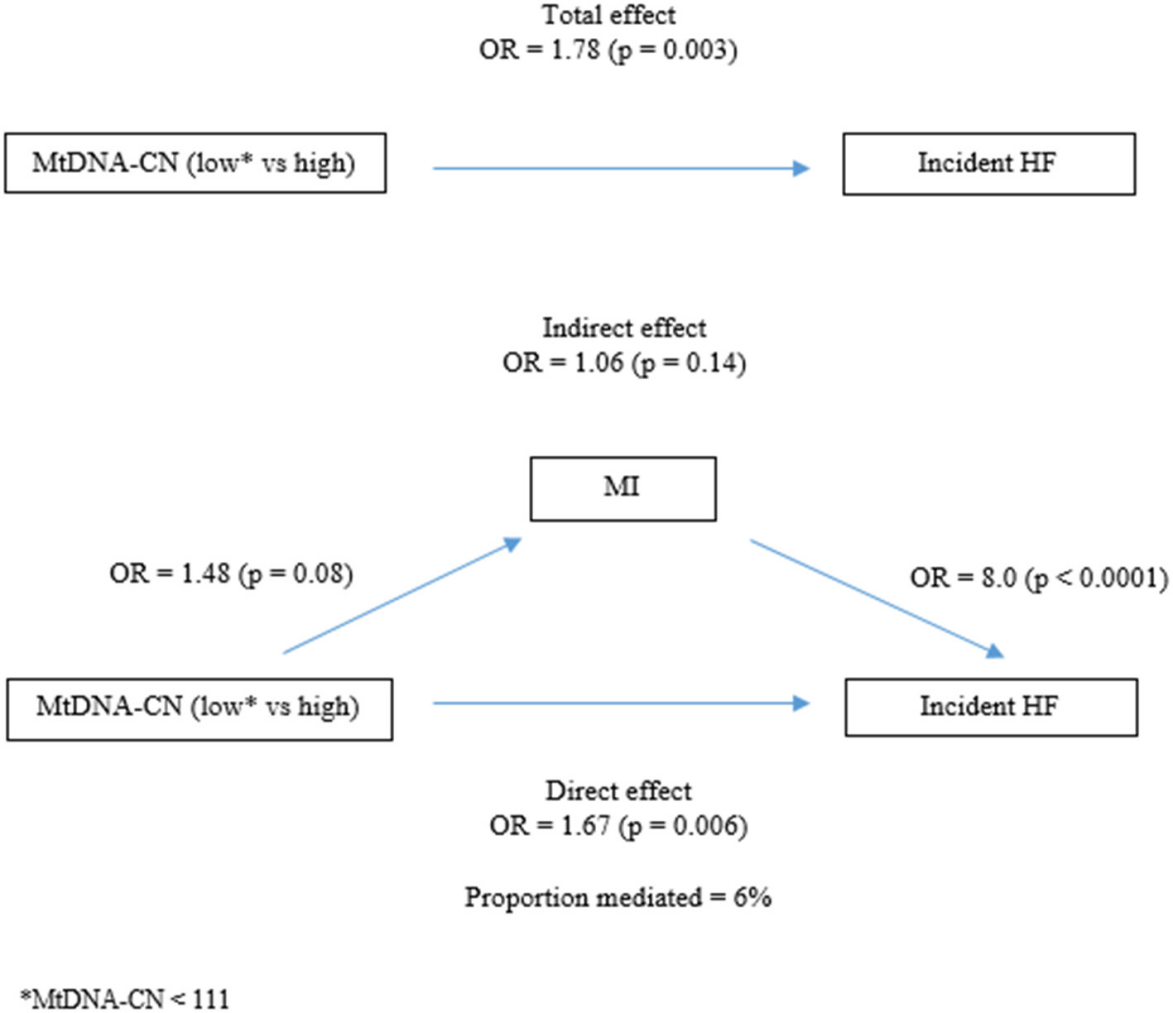
Mediation analysis.

We also investigated whether mtDNA-CN and MI had any confounding, modifying or mediating effect on their associations with incident HF. To disentangle this effect we analyzed potential confounding, interaction and mediation effects. We found that MI slightly reduced the effect of mtDNA-CN on HF, and therefore controlled for MI in the analyses of the association between mtDNA-CN and HF. Neither mtDNA-CN nor MI had any modifying effect on the association with incident HF (interaction term analysis). The mediation analysis showed that although mtDNA-CN affects the risk of MI (OR = 1.48) and MI *via* a risk of future HF (OR = 8.0), the indirect effect of MI on the association between mtDNA-CN and HF was small and did not reach statistical significance (OR = 1.06; *p*-value = 0.14) (Supplementary Table 1 and Figure 3).

### Baseline MtDNA-CN and risk of overall and HF related mortality

During the follow-up, 195 deaths from all causes (overall mortality) and 11 deaths related to HF were observed. In multivariable models adjusted for potential confounders, lower baseline mtDNA-CN (1 SD decrease) was associated with significantly higher risk of overall mortality (HR = 1.20; 95% CI = 1.03–1.39). Similar results were observed when mtDNA-CN was categorized into quartiles (Table 5). Lower baseline levels of mtDNA-CN were also associated with higher risks of HF related mortality (HR = 1.93; 95% CI= 1.04–3.60); however, the statistical significance was lost when adjusted for potential confounders (HR = 1.57; 95% CI = 0.85–2.90), Table 5). The proportionality assumption for the model examining HF mortality was not violated (*p*-value = 0.52).

**Table 5:** Cox regression models examining effect of mtDNA-CN on overall mortality and mortality due to heart failure.

	**Univariate**			**Adjusted** ^a^		
**Outcome: time to death (any)^b^**	**HR**	***p*** **-value**	**95% CI**	**HR**	***p*** **-value**	**95% CI**
MtDNA-CN (decrease, std)^c^	1.27	0.001	1.10; 1.46	1.20	0.02	1.03; 1.39
MtDNA-CN quartiles^d^						
Q_3_ vs. Q_4_	2.02	0.001	1.35; 3.03	1.87	0.004	1.23; 2.85
Q_2_ vs. Q_4_	1.77	0.007	1.16; 2.68	1.50	0.07	0.97; 2.33
Q_1_ vs. Q_4_	2.22	0.001	1.39; 3.55	1.87	0.01	1.14; 3.05
						
Outcome: time to death due to heart failure^e^						
MtDNA-CN (decrease, std)	1.93	0.04	1.04; 3.60	1.57	0.15	0.85; 2.90

a Adjusted for age, smoking, alcohol consumption, education, activity and HDL.

b Number of deaths = 195.

c MtDNA-CN has been reversed and standardized (HR for a one standard deviation decrease in mtDNA-CN).

d Q_1_ = 32.4–88, Q_2_ = 89–110, Q_3_ = 111–130, Q_4_ = 131–226.

e Number of deaths due to heart failure = 11 (found in death register, all causes ICD10 = I50 or I110).

### Sensitivity analyses

Since the number of prevalent MI differed between women with incident and no incident HF (Table 1), we also performed a sensitivity analysis to examine if our results were robust. After exclusion of prevalent MI, the associations between mtDNA-CN and HF in Tables 3–5 only showed minor changes when women with prevalent MI were excluded (data not shown). Thus, the sensitivity analysis further confirmed that our results were robust, and our conclusions remain the same.

## Discussion

Lower mtDNA-CN at baseline is associated with higher risk of HF incidence, independent of potential confounders in a well characterized cohort of middle-aged women followed for a median follow-up of 17 years. Furthermore, we also showed that low mtDNA-CN at baseline was associated with both overall and HF mortality.

HF is a debilitating disease associated with higher morbidity and mortality both in men and women (27). Due to the complexity of the disease conventional risk factors may not accurately predict HF and identification of non-conventional biomarkers which can easily be quantified can assist in better prediction of HF (28). Incidence of HF differ according to sex and is attributed to differences in pathophysiology, risk factors, age and cardiac ejection fraction (29). HF with preserved ejection fraction is more common in women than in men and accounts for at least half of the cases of HF in women (30). Furthermore, women with HF may have a higher probability of HF related readmission than men (3). Levels of mtDNA-CN has been suggested as a biomarker of MI incidence, a major risk factor for HF, in a previous study of ours on middle-aged women (11) and by other researchers in both men and women (31). Most cardiac biomarkers used today do not take the sex differences into account. Therefore, sex-specific reference ranges for cardiac biomarkers used routinely in clinical practice has been proposed (32). Considering the important role of mitochondria in normal functioning of cardiomyocytes, it is not surprising that mitochondrial dysfunction may lead to abnormal functioning of cardiomyocytes, which may eventually result in HF. In agreement with our results, a follow-up study of both men and women showed an inverse association between mtDNA-CN and HF incidence (21). Another study of both men and women and a relatively shorter follow-up of 17 months also showed an inverse relationship between mtDNA-CN and HF risk (20). However, none of the above studies had a stratification according to sex; therefore, it is difficult to conclude whether mtDNA-CN had similar effects on both men and women. Nevertheless, our results demonstrate that lower baseline mtDNA-CN may be a risk factor for HF incidence in middle-aged women.

To evaluate the prognostic performance of a biomarker for an event of interest, it is important to consider potentially competing events whose occurrence could preclude the primary event of interest (33). For example, as shown also in this study, MI is one of the major risk factors of HF (34) and we have previously shown that lower mtDNA-CN is associated with future risk of MI in women (11); hence, can be a competing risk. Furthermore, hypertension and T2D play an important role in the pathophysiology of coronary artery disease more in women than in men; thus, they also are direct or indirect important risk factors of HF in women (2, 35). In our study, mtDNA-CN levels were strongly associated with both systolic blood pressure and T2D. We calculated the cumulative incidence by competing risk regression analysis, where cancer, MI and death were considered competing events. Our results demonstrated that mtDNA-CN is associated with HF incidence independent of the competing risks included in the model. Although MI is a major risk factor for HF, not all MI patients develop HF. Considering the association of mtDNA-CN with both MI and HF incidence, we hypothesized that mtDNA-CN may be a mediating factor between MI and HF or that MI could be a mediating factor between mtDNA-CN and HF. However, we could not find any mediating effect of mtDNA-CN on the association between MI and HF. The mediating role of MI in the association between mtDNA-CN and HF was small and non-significant.

MtDNA-CN is associated with all-cause mortality (36) and higher cardiovascular disease related mortality (20), which is consistent with our study. One of the possible explanations for this association is that the changes in mtDNA-CN influence nDNA methylation at specific loci and result in differential expression of specific genes that may impact disease and mortality *via* altered cell signaling (37). However, the number of deaths due to HF in this study was quite low (*n* = 11) and therefore, this needs to be confirmed in future studies.

### Strength and limitations

This study has several strengths and limitations that must be recognized in the interpretation of our results. The main strength of the study is that it is based on a well characterized population-based follow-up cohort of middle-aged women where an absolute quantification of mtDNA-CN was quantified by a well optimized ddPCR method. Moreover, all diagnoses were collected from a questionnaire and/or Swedish health registers, which provided almost complete information on diagnoses during a long follow-up. We did not have the information on ejection fraction of the participants included in this study, which precluded our possibilities to conduct separate analyses for the different types of HF. Finally, mtDNA-CN was measured only at baseline as follow-up samples were not collected; therefore, we do not know the status of mitochondrial function at the time of diagnosis.

In conclusion, our results demonstrate that mtDNA-CN, an easily quantifiable biomarker in blood, is a molecular risk factor for incident HF, independent of potential confounders and competing events.

## Data availability statement

The original contributions presented in the study are included in the article/Supplementary material, further inquiries can be directed to the corresponding authors.

## Ethics statement

The studies involving human participants were reviewed and approved by the regional Ethical Committee at Lund University approved the study (approval nos. 95/174, 2011/494 and 2015/6) and written informed consent was given by all the participants in the study after full explanation of the purpose and nature of all procedures.

## Author contributions

KS, JS, and AM conceived, designed the study, performed the data analysis, and interpretation. KS and KP performed the statistical analysis. AM and JS collected the samples and clinical data. KS and AM wrote the first draft. KS, JS, KP, and AM revised the article, and approved the final version. All authors contributed to the article and approved the submitted version.

## Funding

This work was supported by grants from Swedish Heart-Lung Foundation, Agreement for Medical Education and Research (ALF) funding from Region Skåne and the Swedish Research Council grants awarded to KS.

## Conflict of interest

The authors declare that the research was conducted in the absence of any commercial or financial relationships that could be construed as a potential conflict of interest.

## Publisher's note

All claims expressed in this article are solely those of the authors and do not necessarily represent those of their affiliated organizations, or those of the publisher, the editors and the reviewers. Any product that may be evaluated in this article, or claim that may be made by its manufacturer, is not guaranteed or endorsed by the publisher.
