# Supplementary material for: Baseline mitochondrial DNA copy number and heart failure incidence and its role in overall and heart failure mortality in middle-aged women
Source: Front Cardiovasc Med. 2022 Nov 10;9:1012403. doi: 10.3389/fcvm.2022.1012403 (PMC9685522; doi:10.3389/fcvm.2022.1012403)
Supplement: Supplementary file 1 [file Table_1.pdf]

SUPPLEMENTARY MATERIAL

Supplementary Table 1. Regression models examining the role of mtDNA-CN and MI in the association to risk of heart failure.

Outcome: incident heart failure	HR/OR† (95% CI)
Univariate analysis	
MI, yes vs no[a]	17.0 (10.9; 26.5)
MtDNA-CN (decrease, std)[b]	1.44 (1.20; 1.74)
Confounding analysis	
Adjusted models:	
MI, yes vs no[a]	16.0 (10.3; 25.1)
MtDNA-CN (decrease, std)[b]	1.39 (1.16; 1.68)
Interaction analysis	
MtDNA-CN as an effect modifier in the association between MI and HF:	
MI, yes vs no[a]	17.5 (9.9; 30.9)
MI*mtDNA-CN (interaction term)	0.84 (0.34; 2.08)
MI as an effect modifier in the association between mtDNA-CN and HF:	
MtDNA-CN (decrease, std)[b]	1.71 (1.18; 2.47)
MI*mtDNA-CN (interaction term)	1.22 (0.12; 12.1)
Mediation analysis	
MtDNA-CN as a mediator between MI and HF:	
Prevalent MI, yes vs no[c]	13.9† (3.9; 50.1)
Indirect effect[d]	1.05† (0.79; 1.39)
MI as a mediator between mtDNA-CN and HF:	
MtDNA-CN (low vs high)[e]	1.78† (1.23; 2.58)
Indirect effect[e]	1.06† (0.98; 1.15)

[a]MI was used as a time-varying covariate (yes = prevalent MI or incident MI (but not after HF), no = no MI at all or time before incident MI or incident MI diagnose after HF. Cox regression model used with time to HF as outcome, HR estimated.
[b]MtDNA-CN has been reversed and standardized (HR for a one standard deviation decrease in mtDNA-CN)
[c]Exposure is prevalent MI and outcome is incident HF yes/no. Logistic regression model used and OR estimated.
[d]Effect of prevalent MI on HF mediated by mtDNA-CN
[e]Exposure is mtDNA-CN (< 111 vs >= 111) and outcome is incident HF yes/no. Logistic regression model used and OR estimated.
[f]Effect of mtDNA-CN on HF mediated by incident MI (yes = incident MI before possible HF, no = no MI at all or incident MI after HF).

Quantification of mtDNA copy number by droplet digital PCR

Absolute quantification of mtDNA-CN AND reference nuclear DNA (nDNA) was performed by our well optimized droplet digital PCR based method. Total genomic DNA was extracted from whole blood (200 µL) using QIAamp 96 DNA Blood (Qiagen, Inc., Hilden, Germany). Extracted DNA was frozen at −20 °C for future use. The mtDNA/nDNA content was assessed using specific primers designed to target mitochondrial MT-ND1 (assay ID: dHsaCPE5029120) gene and nuclear eukaryotic translation initiation factor 2C1 (EIF2C1) (assay ID: dHsaCP1000002) gene, respectively. EIF2C1 is also known as argonaute 1, RISC catalytic component (Gene ID: 26523). Probes targeting nDNA were attached with HEX fluorophore whereas mtDNA were attached with FAM and had lowa Black® FQ quencher on all probes. All primer and probes were obtained from Bio-Rad (Hercules, California, USA). Briefly, 1ng DNA from samples, including positive and negative controls were separately pooled in a 20ul multiplex reaction containing primers (900 nM), probes (250 nM), ddPCR Supermix for probes (no UTP, 2X) and 5U/reaction restriction enzyme (HindIII). The plate with reactions was sealed and incubated at room temperature for 20 min to allow restriction enzyme digestion and then loaded into the automated droplet generator to generate droplet followed by end-point PCR. The after-PCR plate was saved overnight at 4 °C to maximum the droplet recovery (19,000-20,000 droplets/reaction). The plate was read on the droplet reader, data were collected and analyzed using QuantaSoft™ Software to calculate the numbers of positive and negative droplets in each sample. The fraction of positive droplets was then fitted to a Poisson distribution to determine the absolute copy number in units of copies/µl. Quality control for every step of our well optimized ddPCR method was stringent, as described previously (15). The inter and intra CV for absolute quantification of mtDNA-CN was 4.2 % and 3.1%, respectively.
